# Use and Safety of Tyrphostin AG17 as a Stabilizer in Foods and Dietary Supplements Based on Toxicological Studies and QSAR Analysis

**DOI:** 10.3390/foods15020350

**Published:** 2026-01-18

**Authors:** Osvaldo Garrido-Acosta, Ramón Soto-Vázquez, Gabriel Marcelín-Jiménez, Luis Jesús García-Aguirre

**Affiliations:** 1 Facultad de Estudios Superiores Zaragoza, UNAM, Carrera de Química Farmacéutico Biológica, Mexico City 09230, Mexico; 2 Academia de Farmacología y Toxicología, Facultad de Estudios Superiores Zaragoza, UNAM, Mexico City 09230, Mexico; ramonsv@unam.mx; 3 Facultad de Estudios Superiores Zaragoza, UNAM, Carrera de Química Farmacéutico Biológica, Planta Piloto, Mexico City 09230, Mexico; 4 Pharmet S.A. de C.V. (PHARMOMETRICA), Mexico City 02230, Mexico; gabmarcelin@pharmometrica.com.mx; 5 Scire Clinical Solutions SCS, Mexico City 11510, Mexico

**Keywords:** tyrphostin, food stabilizer, food safety, supplement safety, QSAR analysis

## Abstract

This study evaluated two formulations of L-carnitine, which were developed and impregnated in an oil-based self-emulsifying system (SEDDS), the first with tyrphostin AG17 and the second without the addition of tyrphostin AG17. The formulation with tyrphostin AG17 showed the presence of stable microvesicles up to 498 h after its preparation. To establish a robust safety profile in compliance with modern regulatory frameworks and the 3Rs principle (replacement, reduction, and refinement), a toxicological evaluation was conducted integrating an in silico quantitative structure–activity relationship (QSAR) analysis with confirmatory in vivo subchronic toxicity studies. The QSAR analysis, performed using the OECD QSAR Toolbox and strictly adhering to Organization for Economic Co-operation and Development (OECD) validation principles, predicted an acute oral LD50 of 91.5 mg/kg in rats, a value showing high concordance with the historical experimental data (87 mg/kg). Furthermore, computational modeling for repeated-dose toxicity yielded a no-observed-adverse-effect level (NOAEL) of 80.0 mg/kg bw/day, a no-observed-effect level (NOEL) of 60.4 mg/kg bw/day, and an ADI = 56 mg/day. These computational findings were substantiated by a 90-day subchronic toxicity study in male Wistar rats, where daily intragastric administration of tyrphostin AG17 at doses up to 1.75 mg/kg resulted in not statistically significant hematotoxic activity (*p* < 0.05), with a maximum cumulative dose over 90 days of 157.5 mg/kg. Collectively, these data indicate that tyrphostin AG17 combines high stabilizing efficacy with a manageable safety profile, supporting its proposed regulatory status as a functional food additive. Based on these results, it is concluded that tyrphostin AG17 shows promising characteristics for use as a stabilizer in food and other substances.

## 1. Introduction

Recent trends in the food sector indicate consumers’ increased preference for improved safety, transparency, and health benefits. In this context, functional foods have gained attention. Functional foods, which are compounds of varying chemical nature, can exert basic nutritional benefits [1,2]. Emulsions represent an excellent option for food and pharmaceutical products [3,4]. Emulsions are oil droplets dispersed in an aqueous phase, stabilized by emulsifying agents that enhance their stability [5]. Classical emulsifying agents are amphiphilic compounds that adsorb at interfaces, reducing interfacial tension and creating a protective layer; these include surfactants, solid particles, proteins, polysaccharides, and phospholipids [6,7]. Physicochemical factors such as the pH, temperature, and concentration play a crucial role in the stability of emulsions [5,8]. Nanoemulsions encapsulate hydrophobic substances, characterized by a small droplet size, enhanced physical stability of lipophilic compounds, and reduced gravitational separation [9]. Emulsions can encapsulate and protect a variety of nutritional, bioactive, cosmetic, and other substances from local conditions after administration and can also control their release [2,8].

Tyrphostins are a compound family with diverse properties and possible applications in areas such as food, cosmetics, and pharmaceutical products. Although tyrphostins have been known since the 1950s, little attention was paid to them until their use in tyrosine phosphorylation was discovered. The first tyrphostins synthesized were benzene malononitrile tyrphostins. Others were then generated with the basic structure and two or more added aromatic rings and nitrogen atoms, such as AG 1478 and AG 1295 [10].

Tyrphostin AG17 (3,5-di-tert-butyl-4-hydroxybenzylidene-malononitrile, or tyrphostin A9, CAS: 10537-47-0) is a yellow solid, stable in environmental conditions, with an oral LD50 of 87 mg/kg (rat) and a cutaneous LD50 of 300 mg/kg [11]. Previously, because of the chemical structure of tyrphostin AG17, a self-assembled system of Tyrphostin AG17 as a stabilizer was designed and tested to determine the stability of an emulsion of L-carnitine. Self-assembled colloidal delivery systems have proved to be efficient as solubilization and stabilization media. Colloids, polymers, and surfactants, with the capacity to self-assemble into complex hierarchical geometries (micelles, microemulsions, mixed micelles, etc.) in nano spaces, are considered building blocks of self-assembled systems. These systems can to solubilize within small distances and to cause segregation to avoid destructive and undesirable interactions [12,13].

In the contemporary regulatory landscape, the safety assessment of food additives is undergoing a paradigm shift towards predictive toxicology. QSAR models are valuable for prioritization and filling data gaps but are rarely used alone for regulatory decisions. This study integrates QSAR with in vivo studies for a weight-of-evidence approach, following OECD and EFSA recommendations. Regulatory bodies such as the European Food Safety Authority (EFSA) and the U.S. Food and Drug Administration (FDA) increasingly endorse the use of New Approach Methodologies (NAMs), including quantitative structure–activity relationship (QSAR) models, to reduce the reliance on animal testing (the 3Rs principle) and provide mechanistic insights into toxicity [14]. QSAR analysis is based on the fundamental principle of chemistry that the molecular structure determines the physicochemical properties and biological activity. By mathematically modeling the relationship between the structural descriptors (e.g., molecular weight, electronic energy levels, hydrophobicity) and biological endpoints (e.g., LD50, NOAEL) within a “training set” of chemically similar compounds, QSAR can predict the toxicity of a novel substance with high accuracy. For tyrphostin AG17, QSAR analysis offers a critical advantage: it allows for the prediction of chronic toxicity endpoints (which require long-term expensive animal studies) by leveraging data from structurally analogous compounds such as hindered phenols and nitriles. To ensure regulatory validity, the QSAR analysis presented in this manuscript adhered strictly to the OECD principles for the validation of (Q)SAR models [15]:

⮚ Defined endpoint: Acute oral LD50, chronic NOEL, chronic NOAEL, and ADI were defined as the specific endpoints.
⮚ Unambiguous algorithm: Transparent multilinear regression models were used within the OECD QSAR toolkit.
⮚ Defined domain of applicability: We rigorously verified that AG17 falls within the physicochemical limits of the training sets.
⮚ Goodness-of-fit and robustness: Statistical parameters (R^2^, Q^2^) were reported to demonstrate the reliability of the model.
⮚ Mechanistic interpretation: Selected descriptors (e.g., LUMO energy, Log K_OA_) were linked to known mechanisms of AG17 (electrophilicity, membrane partitioning).
⮚ By integrating these high-fidelity in silico predictions with targeted in vivo validation, this study provides a “weight of evidence” (WoE) assessment that is both scientifically robust and ethically responsible.

The aim of this work was to evaluate the safety of tyrphostin AG17 used as an emulsion stabilizer, based on its properties and capacity to create self-assembled systems, and to offer an alternative in the search for solutions in the use of new stabilizers in food, cosmetics, pharmaceutical formulations, and other substances, such as L-carnitine.

## 2. Materials and Methods

Tyrphostin AG17 was synthesized in a synthesis laboratory (IIQUIAP-KAMBERG^®^); it was compared against the reference substance (Tyrphostin A9 from Sigma-Aldrich (Saint Louis, MO, USA), CAS-No: 10537-47-0, Product Number: T182) and certified by ARLEX^®^ laboratories. Solvents and reactants were acquired from recognized providers.

Tyrphostin AG17 as a stabilizer agent.

To evaluate the effect of tyrphostin as a stabilizer agent, two solid formulations with carnitine were developed and impregnated in an oil-based self-emulsifying system (SEDDS). The formulation and stability tests of vesicles were conducted through AVIGAM FARMA^®^. The components of the formulations are presented in Table 1.

To evaluate the stability of the two emulsions, they were poured into synthetic gastric fluid (SGF); the vesicle formation was evaluated, as well as the stability (formed vesicles) at 1, 4, and 498 h.

Tyrphostin AG17 toxicity test.

In accordance with the 3Rs principle in animal research, which involves reducing the number of animals used in research to the minimum necessary and following bioethical standards to minimize animal suffering, and considering the main objective of this research—to evaluate the use of tyrphostin AG17 as a stabilizer in food and food supplements (emulsions)—a test was conducted to determine the hematotoxicity. The study evaluated the hematic biometrics after 90 days of Tyrphostin AG17 administration as part of the subchronic toxicity studies; this test, conducted at the Instituto Politécnico Nacional (IPN) and UNIPREC laboratories, aimed to determine the safety of tyrphostin AG17 by estimating the LD50. Any significant variation in hematic biometrics, due to its proposed use as a stabilizer in food and dietary supplements, would have been sufficient reason to discard it.

For the hematotoxicity test, male Wistar^®^ rats weighing 275 g ± 25 g were used. Before the experiment, these animals were acclimatized in a vivarium for 15 days under standard conditions (22 ± 2 °C, 55 ± 5% humidity, and a 12:12 h light/dark cycle). Food (rat chow) and drinking water were provided ad libitum. The animals (rats) were deprived of food for 4 h prior to the experiment. The use of experimental animals for the toxicological test was carried out in accordance with the applicable regulations and under the authorization of the institutional ethics and bioethics committees.

Tyrphostin AG17 was prepared as an emulsion for intragastric administration using isotonic saline solution (ISS) as the aqueous phase, soybean oil as the oil phase, and Tween 80^®^ as the emulsifying agent. The procedure for preparing the tyrphostin AG17 emulsion (10 mg/kg) for the hematotoxicity test was as follows.

First, 100 mg of tyrphostin AG17 was weighed and poured into an amber Eppendorf tube, to which 5 mL of ISS was added. Then, 100 microliters of Tween 80^®^ were added, and the mixture was sonicated in an ultrasonic bath for 1 min. Next, 5 mL of soybean oil was added; then, the mixture was sonicated again in an ultrasonic bath for 1 min and shaken in a vortex at maximum speed for 30 s.

To evaluate the potential hematotoxic effect using hematic biometry as part of the subchronic toxicity test, tyrphostin AG17 was administered daily for 90 days using a stainless steel 16-gauge cannula for rats. Five groups of ten rats each were formed: one control group, one vehicle group, and three tyrphostin AG17 groups (0.175, 0.55, and 1.75 mg/kg). For the hematic biometry, blood was drawn from the rats’ abdominal artery using a syringe and collected in tubes containing anticoagulant for further analysis. Statistical tests were performed using SigmaPlot 12.0 Exact Graphs and Data Analysis software (V.10.2), Systat Software Inc. (Palo Alto, CA, USA).

LD50 by QSAR analysis.

In accordance with the 3Rs principle for animal research, for the replacement principle, the LD50 was obtained through QSAR analysis at the Facultad de Química de la Universidad Nacional Autónoma de México [Chemistry Faculty, UNAM]; this can be adopted as a reference for future research studies. The procedure and characteristics for this study were as follows.

Oral LD50 data in rats were obtained for 5607 organic compounds from an initial categorization, which were defined based on their structural similarity (e.g., the presence of hindered phenol groups, nitrile functionalities, and benzenoid rings) and mechanistic similarity (uncouplers, phenols), resulting in 48 analogous compounds for the training set, each with its CAS number. The reported doses and data for the 48 compounds were obtained from the Hazard Evaluation and Support System database (HESS). This database classifies chemical groups that may produce similar toxic effects upon repeated oral administration. The HESS database contains experimental information on more than 500 compounds, grouped into 33 categories according to their mechanism of action and organ-specific effects (e.g., hepatotoxicity, hemolytic anemia). For tyrphostin AG17, no structural alerts were identified in any of the 33 categories of the HESS database. Consequently, based on the QSAR analysis and the data source for the test, no significant toxic effects were anticipated from the subchronic administration (up to 90 days), according to the structural similarity principles of this database.

The statistical model used in this study has proven to be highly accurate and consistent in determining the LD50 in the rat model oral route. The model’s accuracy and reliability result from the careful selection of molecular descriptors that best represent the properties of the chemical compounds in question and the rigorous validation of the model with experimental data. Furthermore, the model was designed to include only compounds within its domain, ensuring that the predictions were accurate and reliable for these specific compounds.

For this study, we used the OECD QSAR toolbox program version 3.4.0.17 (MOPAC version 7.0), with version 3.8.8/3.1.2 of its database [16,17,18,19,20,21,22,23,24].

Due to this this method needing to evaluate the applicability domain (AD), to ensure the reliability and accuracy of QSAR predictions, all composites or predictions based on descriptors outside this domain were excluded from the model, thus ensuring its robustness.

Chronic Toxicity Models (NOEL and NOAEL)

The primary purpose of profilers within the QSAR Toolbox is to categorize chemical substances by extrapolating experimental data to formulate QSAR equations for specific targets. In this study, the calculated endpoints included the No Observed Effect Level and No-Observed-Adverse-Effect Level (NOEL and NOAEL). To identify the optimal prediction equations, all descriptors were evaluated, prioritizing statistical validity (where R2 > 0.8 and Q2 > 0.6) and ensuring the values remained within the profiling domain.

Acceptable Daily Intake (ADI) Derivation

The ADI was derived from the predicted NOAEL using the standard safety factor approach mandated by the JECFA (Joint FAO/WHO Expert Committee on Food Additives) and EFSA [25].

(1) $$ADI=\frac{NOAEL}{SF}$$

Safety Factor (SF): a composite factor of 100 was applied.

Ten times for interspecies variability (extrapolating from rat to human).

Ten times for intraspecies variability (accounting for sensitive human subpopulations such as children or the elderly).

## 3. Results

Tyrphostin AG17 as stabilizer agent.

As shown in Table 2, the standard formulation without tyrphostin AG17 rapidly failed in the Simulated Gastric Fluid (SGF). The oil phase separated, which would prevent passive absorption and lead to uncontrolled carnitine release. In contrast, the formulation stabilized with tyrphostin AG17 maintained vesicle integrity even after 498 h, demonstrating that it is a viable formulation for oral administration.

**Figure 1:**
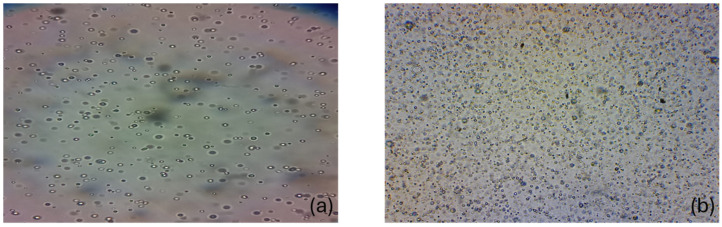
Microscopic photographs (40X) of the vesicles that self-assemble in the SGF and their behavior over time. (**a**) The vesicles after 60 min in gastric fluid; (**b**) the vesicles stable at the sampling time of 240 min.

After 10 h, the formulation with tyrphostin AG17 in SGF contained vesicles capable of continuing to release their contents (carnitine), while the formulation without tyrphostin AG17 had already separated into two phases (Figure 1).

Subacute test of hematotoxicity (hematic biometry).

**Figure 2:**
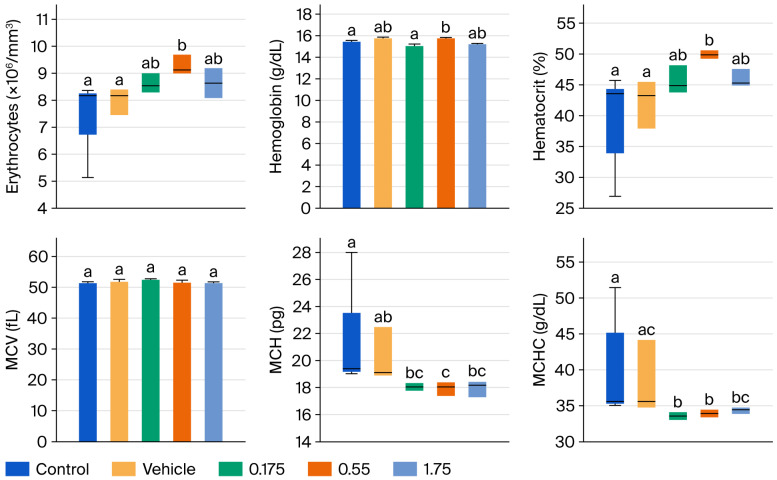
Effect of subchronic tyrphostin AG17 administration for 90 days on hematic biometry values in male Wistar rats (part 1). a: Control; b: Vehicle; c: tyrphostin AG17 0.175 mg/kg. Mean + standard error plotted; one-way ANOVA statistical test and SNK post hoc test, with a statistically significant difference *p* > 0.05.

**Figure 3:**
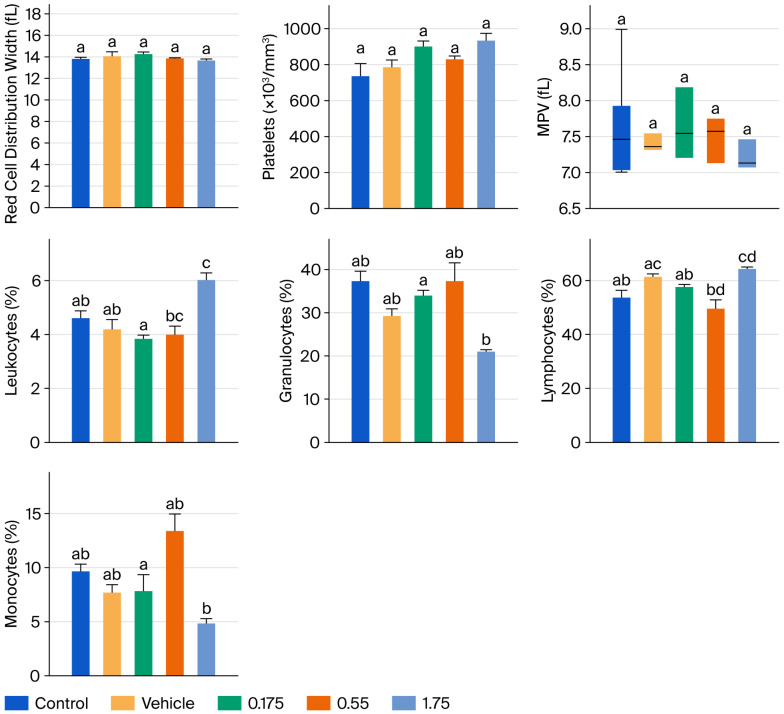
Effect of subchronic tyrphostin AG17 administration for 90 days on hematic biometry values in male Wistar rats (part 2). a: Control; b: Vehicle; c: tyrphostin AG17 0.175 mg/kg; d: 0.55 mg/kg. Mean + standard error plotted; one-way ANOVA statistical test and SNK post hoc test, with a statistically significant difference of *p* > 0.05.

Figure 2 and Figure 3 present the results of hematic biometry. There are no significant differences between the groups administered the tyrphostin AG17 emulsion (treated groups) and the control groups in the variables of mean corpuscular volume (MCV), platelet distribution width, platelets, and mean platelet volume (MPV). However, there are statistically significant differences in erythrocytes, hematocrit, MCH, and MCHC, as well as in leukocytes, granulocytes, lymphocytes, and monocytes, but these differences are not outside the acceptable reference ranges. Therefore, this indicates that there is no impairment of bodily function after 90 days of daily administration of various doses of the emulsion formulated with tyrphostin AG17 (0.175, 0.55, and 1.75 mg/kg per day).

QSAR toxicity predictions

The computational models provided a detailed toxicological profile for AG17, covering both acute hazards and chronic safety limits.

Acute Oral Toxicity (LD50)

The reported results of the QSAR analysis were based on the Annex I (Q)SAR model reporting format (QMRF) v.2.1 from the (Q)SAR Assessment Framework: Guidance for the regulatory assessment of (quantitative) Structure–Activity Relationship models and predictions (2nd edition) from Series on Testing and Assessment No. 405 [26].

QSAR identifier

Model tyrphostin 9 LD50.

Summary

The toxicity of the target chemical (91.5 mg/kg) is predicted by the QSAR “Model Tyrphostin 9 LD50”.

The target chemical falls within the applicability domain of the prediction.

Date. This analysis was performed on 31 October 2025.

Availability of information about the model.

The statistical model used in the study has proven to be highly accurate and consistent in predicting the LD50 in rats via the oral route. The model’s accuracy and reliability result from the careful selection of molecular descriptors that best represent the properties of the chemical compounds in question and the rigorous validation of the model with experimental data. Furthermore, the model was designed to include only compounds within its domain, ensuring that the predictions were accurate and reliable for these specific compounds.

OECD Principle 1: Defined end point (Table 3).

OECD Principle 2: “An unambiguous algorithm”.

The equation used was as follows:

LD50 = +1.10 (±0.14) + 8.92 × 10^−4^ (±2.00 × 10^−4^) × FM reaction soil [kg/h] −0.0799 (±0.0208) × Log K_OA_ (Henry’s law constant model) −2.03 × 10^−4^ (±7.43 × 10^−4^) × melting point (adapted Joback method) [°C] + 3.55 × 10^−3^ (±2.35 × 10^−3^) × molecular weight [Da] − 0.0110 (±0.0357) × number of heavy atoms + 23.9 (±6.4) × relative number of N atoms, log(1/mol/kg). (2)

OECD Principle 3: A defined domain of applicability.

The target chemical falls within the applicability domain.

(1) Parametric boundary: The FM reaction soil of the target chemical should be ≥−0.936 kg/h.

(2) Parametric boundary: The FM reaction soil of the target chemical should be ≤1.9 × 10^3^ kg/h.

(3) Parametric boundary: The log K_OA_ (Henry’s law constant model) of the target chemical should be ≥0.923.

(4) Parametric boundary: The log K_OA_ (Henry’s law constant model) of the target chemical should be ≤45.7.

(5) Parametric boundary: The melting point (adapted Joback method) of the target chemical should be ≥−125 °C.

(6) Parametric boundary: The melting point (adapted Joback method) of the target chemical should be ≤350 °C.

(7) Parametric boundary: The molecular weight of the target chemical should be ≥71 Da.

(8) Parametric boundary: The molecular weight of the target chemical be ≤1.18 × 10^3^ Da.

(9) Parametric boundary: The number of heavy atoms in the target chemical should be ≥4.92.

(10) Parametric boundary: The number of heavy atoms in the target chemical should be ≤85.1.

(11) Parametric boundary: The relative number of N atoms in the target chemical should be ≥−3.57 × 10^−5^.

(12) Parametric boundary: The target chemical should have a value of relative number of N atoms ≤0.049.

OECD Principle 4: Appropriate measures of goodness-of-fit, robustness, and predictivity.

**Figure 4:**
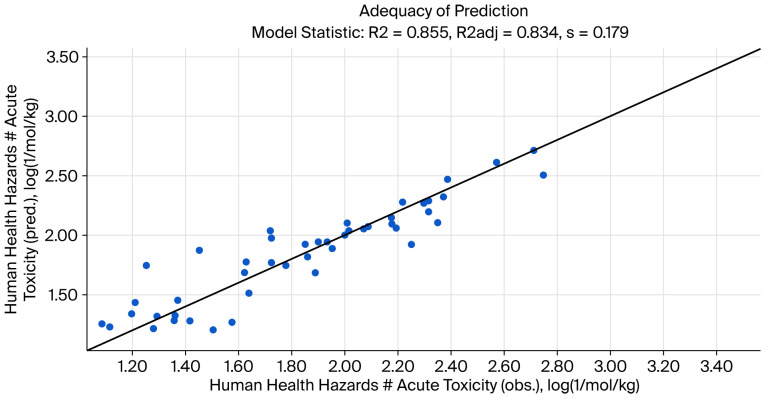
Adequacy of prediction from QSAR analysis “DL50 tyrphostin AG17 oral rat”.

**Figure 5:**
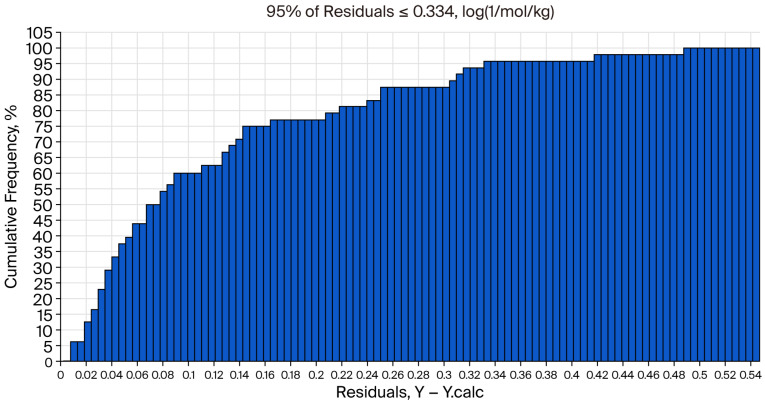
Cumulative frequency from QSAR analysis “DL50 Tyrphostin AG17 oral rat”.

Figure 4 shows the adequacy of the prediction, and Figure 5 shows the cumulative frequency from the QSAR report.

- Prediction range:

31.6–265 mg/kg (95.0% confidence).

- Statistics of the prediction model:

N = 48; count of data points.

R2 = 0.855; coefficient of determination.

R2adj = 0.834; adjusted coefficient of determination.

Q2 = 0.784; coefficient of determination by “leave-one-out” validation.

SSR = 1.31; sum of squared residuals.

s = 0.179; sample standard deviation of residuals.

F = 40.3; Fisher function.

Fa = 2.74; Fisher threshold for statistical significance (95.0% confidence).

OECD Principle 5: A mechanistic interpretation, if possible.

Profiling results for the target substance:

OECD HPV Chemical Categories:

Not categorized.

- Substance type:

Discrete chemical.

US-EPA New Chemical Categories: 

Phenols (acute toxicity).

Chemical Elements: Group 14—Carbon C, Group 15—Nitrogen N, and Group 16—Oxygen O.

- Groups of elements:

Non-metals.

- Lipinski Rule Oasis: s

Bioavailable.

- Organic Functional Groups: Alkene, alkyl arenes, aryl, malononitrile, nitrile, phenol, and tert-butyl/
- Organic Functional Groups (Nested): Alkene, alkyl arenes, malononitrile.
- Overlapping groups: phenol.

Organic Functional Groups (US EPA): Acetylenic carbon [#C], alcohol, olefinic attach [-OH], aliphatic carbon [CH], aliphatic carbon [-CH2-], aliphatic carbon [-CH3], aromatic carbon [C], dicyano [=C(C#N)C#N], dicyano [C=C(C#N)C#N], dicyano [N=C(C#N)C#N], hydroxy, aromatic attach [-OH], olefinic carbon [=CH- or =C<], oxygen, one aromatic attach [-O-], tertiary carbon.

Norbert Haider (checkmol):

Aromatic compound, hydroxy compound, phenol.

- Tautomers unstable:

Stable form.

- Repeated dose (HESS):

Not categorized.

The QSAR was model used to predict the compound is statistically robust, lending high reliability to the results obtained. Therefore, the predicted LD50 (oral) value can be used with certainty to meet the classification and labeling requirements for the substance. This value is equally applicable to the assessment of risks to human health and the environment, as well as to the estimation of toxicity in chemical mixtures. The result meets the requirements of Annex J of the FAO Manual, validating its usefulness in the risk assessment of chemical substances. Furthermore, considering that the experimentally reported value is LD50 = 87 mg/kg, cited in the “Special Publication of the Entomological Society of America”, 78-1(21), 1978, we can confirm that the prediction model is sufficiently robust, as it does not exceed the maximum permissible ±10% of the reported value, which in this case is 91.5 mg/kg.

Chronic Toxicity (NOEL and NOAEL)

The repeated-dose toxicity models provided critical values for long-term safety assessments.

NOEL prediction: The model predicted a no-observed-effect level (NOEL) of 60.4 mg/kg/day, which represents the dose at which no effects (adverse or non-adverse) are observable.

NOAEL prediction: The model predicted a no-observed-adverse-effect level (NOAEL) of 80.0 mg/kg/day.

Statistical robustness: The NOAEL model demonstrated excellent statistical fit (R2 = 0.937) and predictive power (Q2 = 0.781).

Descriptor analysis: The QSAR model identified LUMO energy (−1.13 eV) as a critical descriptor. Mechanistically, this specific energy level indicates that AG17 is a “soft” electrophile. This property allows the molecule to effectively delocalize the negative charge of its anionic form (phenolate) across the dicyanovinyl group. This charge delocalization is the rate-limiting factor that allows the anion to traverse the hydrophobic inner mitochondrial membrane (IMM) and return to the intermembrane space to pick up another proton.

Without this specific LUMO configuration, the anion would be trapped in the matrix, breaking the uncoupling cycle. Furthermore, because this mechanism involves reversible proton transport rather than irreversible covalent binding (characteristic of “hard” electrophiles with higher LUMO gaps), it supports the existence of a clear safety threshold (NOAEL) rather than stochastic genotoxicity.

The predicted NOAEL (80 mg/kg/day) is close to the acute LD50 (91.5 mg/kg). This steep dose–response relationship is characteristic of mitochondrial uncouplers. Toxicity in this class is often threshold-based: below the threshold of ATP depletion and hyperthermia, the organism adapts (mild uncoupling); once the threshold is crossed, acute toxicity onset is rapid. This suggests that if exposure remains below the threshold, the safety margin is preserved.

Derivation of Acceptable Daily Intake (ADI)

Using the predicted NOAEL of 80 mg/kg/day as the Point of Departure (PoD):

ADI = (80.0 mg/kg per day)/100 = 0.8 mg/kg bw per day.(3)

Interpretation:

- For an average adult weighing 70 kg, the safe daily intake would be 0.8 X 70 = 56 mg/day.
- This ADI provides a substantial permissible intake for a food additive, which is typically used in milligrams or micrograms of quantities per serving.

In vivo subchronic toxicity validation

The 90-day oral toxicity study in rats provided critical in vivo confirmation of the QSAR predictions.

Hematotoxicity results: The hematic biometry analysis assessed multiple parameters to detect potential systemic toxicity, bone marrow suppression, or inflammatory responses.

Erythrocytes (RBC), hematocrit, and hemoglobin: There were no statistically significant differences (*p* > 0.05) between the AG17-treated groups (0.175, 0.55, 1.75 mg/kg/day) and the control or vehicle groups, which indicates no hemolytic anemia or erythropoietic suppression, a crucial finding given that some phenol derivatives can induce hemolysis.

Leukocytes (WBC) and differential counts: While minor statistical variations were noted in some leukocyte subsets, the values remained well within the physiological reference ranges for the Wistar strain; these fluctuations were not dose-dependent and were interpreted as normal biological variation rather than compound-related toxicity.

Platelets: No significant alterations were observed, ruling out thrombocytopenia.

Survival and general health:

The highest dose administered (1.75 mg/kg/day) resulted in a cumulative dose of 157.5 mg/kg over 90 days; the absence of adverse effects at this level confirms that the daily “safe” threshold is indeed much higher, consistent with the QSAR-predicted NOAEL of 80 mg/kg/day.

## 4. Discussion

The absence of structural alerts for specific organ toxicity (e.g., hepatotoxicity, nephrotoxicity) in HESS database analysis further supports the conclusion that tyrphostin AG17 acts via a functional metabolic mechanism rather than a structural toxic mechanism (such as adduct formation). This explains why the chronic safety profile is favorable despite the acute potency: the body can adapt to mild metabolic modulation (mild uncoupling), whereas it cannot adapt to cumulative structural damage.

The use of tyrphostin AG17 may provide safety and health benefits [8] for new food stabilizers. The amphipathic nature of tyrphostin AG17 gives it the ability to be a good emulsifying agent, since it encapsulates and protects various substances (for example, L-carnitine) from the acidic environment of the stomach [5,6,7,8], providing stability to the microvesicles formed in the emulsion with an observed duration of more than 498 h in the SGF [9,27], which translates into a controlled release of the substance; this represents an advantage for the administration of nutrients, bioactive compounds, and other substances through the gastrointestinal tract (enteral administration) [2,8].

ADI justification and safety margins

The derived ADI of 0.8 mg/kg/day provides a robust safety margin for the use of AG17 as a food additive.

Estimated daily intake (EDI): As a stabilizer in a dietary supplement (e.g., a capsule or tablet), AG17 would likely be used at concentrations of 0.1% to 1% (1000–10,000 ppm).

Consumption of one capsule would deliver 1.0 to 10.0 mg of AG17.

For a 70 kg adult, 10.0 mg represents a dose of 0.14 mg/kg.

Margin of safety (MOS):

Ratio of ADI (0.8) to EDI (0.14) is 5.7.

Ratio of NOAEL (80) to EDI (0.14) is 571. This high margin of safety (nearly 600-fold relative to the no-effect level in animals) far exceeds the standard regulatory requirement of a 100-fold margin. This demonstrates that at technological use levels, AG17 poses negligible risk.

## 5. Conclusions

Vesicle systems that self-assemble in a simulated dissolution medium and are stabilized by tyrphostin AG17 significantly improve L-carnitine permeability. Tyrphostin AG17 is essential for maintaining vesicle integrity in the SGF, not only through a direct effect but also by providing steric stability and forming a robust interfacial complex unaffected by the acidic pH of the SGF.

The use of tyrphostin AG17 as a stabilizer for food and food supplements is safe, as it did not present toxic effects in the studies carried out, with an LD50 of 91.5 mg/mL in the QSAR study performed in this research, and no hematotoxic effect was detected in the hematic biometry after administration of 0.175, 0.55, or 1.75 mg/kg for a period of 90 days, with a maximum cumulative dose of 157.5 mg/kg over 90 days.

Tyrphostin AG17 shows promising qualities as a food and dietary supplement stabilizer, and the results obtained in the acute and subchronic toxicity tests carried out allow us to consider continuing studies and tests with these compounds.

Based on the integrated evidence from the technological performance trials, in silico QSAR modeling, and in vivo subchronic toxicity testing, the following conclusions are drawn:

1. Technological necessity: Tyrphostin AG17 is a highly effective stabilizer for oil-based self-emulsifying systems (SEDDSs), offering a stability performance (498 h in SGF) that is better than other substances to stabilize formulations.
2. Acute hazard characterization: Tyrphostin AG17 has an LD50 of 91.5 mg/kg (GHS Category 3). This necessitates strict occupational safety controls during its manufacture and handling as a raw material but does not restrict its safety as a low-level additive.
3. Chronic safety validation: QSAR models and in vivo studies consistently indicate a high threshold for chronic toxicity (NOAEL 80 mg/kg/day).
4. Safe exposure limits: An ADI of 0.8 mg/kg bw/day has been scientifically established and validated.

Regulatory proposal: It is proposed that tyrphostin AG17 be recognized as safe for use as a food additive (stabilizer/antioxidant) in dietary supplements and functional foods, subject to the following conditions:

- Specification: Purity > 99%.
- Usage Limit: Formulations should be designed such that the Estimated Daily Intake (EDI) does not exceed 0.4 mg/kg/day (e.g., 50% of the ADI, preventively) to ensure a conservative safety margin.
- Labeling: Standard labeling as a food additive/stabilizer; no hazard warning is required on the final consumer product due to the low concentration.

This study supports the submission of a dossier for GRAS (Generally Recognized As Safe) status in the USA or Novel Food authorization in the EU, providing a compelling case for tyrphostin AG17 as a next-generation stabilizer that addresses the stability challenges of modern nutraceuticals.

## Figures and Tables

**Table 1:** Formulations.

Component ^1^	Formulation 1	Formulation 2
Oil-based	Yes	Yes
Surfactant	Yes	Yes
Co-solvent	Yes	Yes
Tyrphostin AG17	Yes	No

^1^ The amounts are confidential information.

**Table 2:** Evaluation of vesicle stability in simulated gastric fluid (SGF).

Formulation	0 h	1 h	4 h	498 h
SEDDS (Without Tyrphostin AG17)	Vesicles formed	Instability, visible aggregation	Phase separation: the oil phase merges, and the emulsion breaks	Two phases are visible, without visible vesicles
SEDDS (With Tyrphostin AG17)	Thin and homogeneous vesicles	Stable vesicles	Stable vesicles	Vesicles are intact and the emulsion is maintained

**Table 3:** Descriptors of model QSAR “Tyrphostin AG17 LD50”.

Descriptor	
FM reaction soil	1.89 + 3 kg/h
Log K_OA_ (Henry’s law constant model)	14.0
Melting point (adapted Joback method)	298 °C
Molecular weight	282 Da
Number of heavy atoms	21.0
Relative number of N atoms	0.0465
Endpoint (dep. variable)	
Human health hazards; acute toxicity	

## Data Availability

The original contributions presented in this study are included in the article. Further inquiries can be directed to the corresponding authors.

## Abbreviations

The following abbreviations are used in this manuscript:

EFSA	European Food Safety Authority
FDA	Food and Drug Administration
HESS	Hazard Evaluation and Support System Database
LD50	Lethal Dose 50
NAMs	New Approach Methodologies
NOAEL	No-Observed-Adverse-Effect Level
NOEL	No-Observed-Effect Level
OECD	Organization for Economic Co-operation and Development
QSAR	Quantitative Structure–Activity Relationship
SEDDS	Self-Emulsifying System
SGF	Simulated Gastric Fluid
